# 

**Published:** 1908-07-25

**Authors:** 


CONTENTS.

| | PAGE |
|---|---|
| **Hospitals and Medical Education** ... ... ... ... | 433 |
| **The Interim Report of the Whisky Commission** | 434 |
| **Annotations**— | |
| The Cause of Sunstroke—The Communion Cup—Government Research ... ... ... ... ... ... ... ... ... | 435 |
| **Medical Opinion and Movement** ... ... ... ... | 436 |
| **Hospital Clinics**— | |
| Cerebral Abscess. By Percy Sargent, F.R.C.S. ... ... ... | 439 |
| The Treatment of Whooping Cough. By Edmund Hughes, M.R.C.S.Eng., L.R.C.P.Lond. ... ... ... ... ... | 442 |
| **Medicine**— | |
| Enlargements of the Spleen—I. ... ... ... ... ... | 443 |
| Hemiplegia after Scarlet Fever ... ... ... ... ... | 444 |
| **Surgery**— | |
| Some Aspects of Intestinal Obstruction ... ... ... ... | 445 |
| **Diseases of Children**— | |
| Bronchiectasis ... ... ... ... ... ... ... ... | 446 |
| **The General Practitioner's Column**— | |
| Tuberculin Treatment in General Practice... ... ... ... | 447 |
| **Dermatology**— | |
| Finsen Light in the Treatment of Lupus Vulgaris ... ... | 448 |
| **Therapeutics**— | |
| Prescriptions and Dispensing ... ... ... ... ... ... | 449 |
| **Mental Diseases**— | |
| Melancholia ... ... ... ... ... ... ... ... ... | 450 |
| **Medico-Legal Points**— | |
| Fees Payable to Medical Men as Witnesses ... ... ... | 451 |
| **New Appliances and Things Medical** ... ... ... | 452 |
| **The Practitioner's Relaxations and Hobbies**— | |
| A Week in a Caravan ... ... ... ... ... ... ... | 453 |
| **Social and Poor-law Problems** ... ... ... ... | 454 |
| **News and Coming Events** ... ... ... ... ... | 455 |
| **Nursing Administration**— | |
| I.—Probationers in Excess ... ... ... ... ... ... | 457 |
| The Common Task ... ... ... ... ... ... ... | 458 |
| **Editor's Letter-Box** ... ... ... ... ... ... | 458 |

